# Cured but not healed: high burden of pulmonary disability and multimorbidity among TB survivors in India

**DOI:** 10.5588/pha.26.0038

**Published:** 2026-08-24

**Authors:** S. Subramanian, P. Thekkur, P. Chinnakali, J.P. Tripathy, G. Ramaswamy, V. Venugopal, I.S. Sehgal, K. Soundappan, P. Naik, D. Nair, S. Malegaonkar, V.M. Babu, K.Y. Jothula, A. Malik, L. Devaraj, M. Rajaram, S. Sarkar, A.D. Harries, R. Zachariah

**Affiliations:** 1 Jawaharlal Institute of Postgraduate Medical Education and Research, Puducherry, India;; 2 Centre for Operational Research, International Union against Tuberculosis and Lung Disease, Paris, France;; 3 UNICEF, UNDP, World Bank, WHO Special Programme for Research and Training in Tropical Diseases (TDR), Geneva, Switzerland;; 4 All India Institute of Medical Sciences, Nagpur, India;; 5 Tufts University School of Medicine, Boston, MA, USA;; 6 All India Institute of Medical Sciences, Bibinagar, India;; 7 All India Institute of Medical Sciences, Deoghar, India;; 8 Post Graduate Institute of Medical Education and Research, Chandigarh, India;; 9 Dahdaleh Institute for Global Health Research, School of Global Health, York University, Toronto, ON, Canada;; 10 Yenepoya Medical College, Yenepoya (Deemed to be University), India;; 11 Independent Researcher, Scottsdale, AZ, USA;; 12 London School of Hygiene and Tropical Medicine, London, UK.

**Keywords:** tuberculosis, post-TB lung disease, multimorbidity, chronic respiratory disease, SORT IT, operational research

## Abstract

**SETTING:**

Five districts across five states of India.

**OBJECTIVE:**

To assess the prevalence of TB-associated disability, comorbidities, risk determinants, and linkage to care among adults completing TB treatment between October 2024 and December 2025.

**DESIGN:**

A cohort study using primary data collection. Trained research staff conducted all the assessments. Pulmonary disability meant impaired lung function on spirometry and/or walking <400 m on 6-minute walk test. Linkage to care was assessed through telephonic follow-up after a month of referral.

**RESULTS:**

Of 780 participants, 85% had at least one comorbidity, risk determinant, or disability (multimorbidity). The prevalence of pulmonary disability was 60% (95% confidence interval: 56–64). Undernutrition (36%), diabetes (24%), and hypertension (19%) were the most common comorbidities, with 13% of diabetes and 55% of hypertension being newly detected. Pulmonary disability was associated with older age, pulmonary TB, undernutrition, mental health disorder, smoking, and silica exposure. Referral ranged from 12% to 100% for various conditions, with low rates for mental health disorders (12%) and recreational drug use (33%).

**CONCLUSION:**

Successful completion of TB treatment and bacteriological cure does not imply restoration of health-related quality of life. Early integration of patient-centred care for multimorbidity is essential for restoring health among TB survivors.

## Graphical abstract

**Figure:**
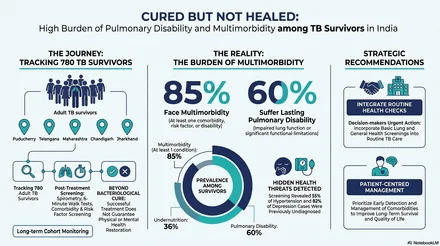

Globally, TB is the leading cause of death from a single infectious agent. In 2024, an estimated 10.7 million people developed TB and 1.23 million died, despite declining incidence and mortality over two decades.^1^ Globally, 85% with TB complete TB treatment and are considered cured of the disease.^1^ However, most continue to suffer from TB-associated disability.^2–5^ Of the estimated 122 million disability adjusted life years lost due to TB, 58 million (47%) are attributed to post-TB sequelae.^6^ Further, people completing TB treatment have higher risk of developing TB disease and all-cause mortality compared with the general population.^7,8^ Recognising this substantial post-TB treatment burden, the Union and its partners along with the 1st International Post-Tuberculosis Symposium recommended the addition of ‘Fourth 90’ to the global ‘90:90:90’ targets, ensuring good health–related quality of life in 90% of those completing treatment.^9,10^ Subsequently, international clinical standards for assessing and managing TB-associated disability were introduced.^11^ The WHO also recommended that national TB programmes (NTPs) should integrate systematic screening to identify and manage TB-associated disability during and at the end of TB treatment.^12^ However, NTPs failed to integrate this due to a lack of ‘know-how’ on TB-associated disability assessment and management.^13^

Recent implementation research studies from China and four countries in Africa demonstrated the feasibility of assessing and managing risk determinants, comorbidities, and pulmonary disability among people completing TB treatment under programmatic conditions.^14–17^ In Africa, about 72% of patients completing TB treatment had at least one risk determinant, comorbidity, or pulmonary disability (inability to walk 400 m in 6-minute walk test [6MWT]) and two thirds of these patients could be referred for further care. However, 80% of those with pulmonary disability required referral outside their original health facility for pulmonary rehabilitation.^17^

India is a high-TB-burden country, contributing to about 30% of the notified TB patients globally.^1^ Previous studies from tertiary care hospitals of India with a smaller sample size and diverse assessment methods have reported a high burden (30%–60%) of pulmonary disability among people completing TB treatment.^4,18–20^ Acknowledging the importance and high burden of pulmonary disability, the National TB Elimination Programme (NTEP) recommended systematic detection and management of pulmonary disability.^11^ However, there is no operational guideline on how to assess pulmonary disability in programmatic settings and it is not currently being implemented.

Thus, we conducted this study among adults completing TB treatment in five districts of India, to assess the prevalence of risk determinants, comorbidities, and pulmonary disability, and to report on linkage to care for those identified with any of these conditions. Additionally, we assessed work absenteeism and wage loss during TB treatment, to understand the socio-economic consequences of TB beyond clinical outcomes.

## METHODS

### Study design

Cohort study using primary data collection.

### Study setting

The study was conducted in five selected districts across five states of India: Puducherry, Yadadri Bhongir (Telangana), Nagpur (Maharashtra), Chandigarh, and Deoghar (Jharkhand). The NTEP is implemented in each district through the district health system, coordinated by district TB officers. TB diagnosis is established through sputum microscopy, nucleic acid amplification test assay, and/or chest radiography, followed by classification into drug-sensitive or drug-resistant TB.^21–23^ Drug-sensitive TB is treated with a 6-month regimen of HRZE followed by HRE,^24^ while drug-resistant TB is managed with individualised regimens for 6–18 months.^25^ Although post-treatment clinical evaluations are recommended, no guideline exists for structured assessment of pulmonary function, risk determinants, and comorbidities under routine programme settings.

### Study population

The study included all adults (aged ≥ 18 years) who completed TB treatment (cured and treatment completed) for any form of TB during October 2024 to December 2025. Patients not consenting or unable to answer were excluded from the study. The minimum sample size was calculated to be 768, assuming 20% prevalence of pulmonary disability, 4% absolute precision, 95% confidence level, and design effect of 2.^16,17^

### Study procedures

Study procedures followed the generic protocol implemented by the Special Programme for Tropical Disease Research (TDR) in China and Africa.^14–17^ However, two major modifications were made: first, TB-associated disability assessments were conducted by dedicated research staff mostly at patients’ residences rather than by health care workers due to operational challenges; and second, handheld spirometry was used alongside the 6MWT to assess pulmonary disability. Research staff in each district were trained in standard operating procedures including 6MWT, spirometry, questionnaire administration, and data entry using EpiCollect5, a mobile-based data capture tool. A weekly list of eligible participants with demographic and TB treatment details was obtained from Ni-kshay, a web-enabled patient management system for TB. Eligible participants were enrolled within 1 month of treatment completion. The following procedures were conducted:

- Questionnaire-based assessment: Details of occupation prior to TB diagnosis, monthly income in Indian rupees, and number of days of absenteeism during the TB treatment are collected. Information on residual symptoms, risk determinants (tobacco use, alcohol use, occupational silica exposure, and recreational drug use in the last month), and comorbidities (HIV, diabetes mellitus [DM], hypertension, and mental health problems) was collected. Probable depression was assessed using the Patient Health Questionnaire-2; scores above 3 were considered ‘probable depression’.^26^ The CAGE (Cut, Annoyed, Guilty, Eye opener) questionnaire was applied to those who reported alcohol use in the last month; a score ≥2 was considered ‘probable alcohol dependence’.^27^
- Physical examination and investigations: Height and weight were measured to calculate body mass index (BMI) for ascertaining undernutrition (BMI <18.5 kg/m^2^); blood pressure was recorded to detect hypertension (systolic ≥140 mmHg and/or diastolic ≥90 mmHg); and random capillary blood glucose was measured to detect hyperglycaemia (≥200 mg/dl).^28,29^
- 6MWT was performed on a flat surface over a measured course of at least 20 m. Participants were instructed to walk for 6 min, with the option to slow down or stop if they became exhausted and to resume when able. The total distance covered was recorded by research staff.
- Spirometry was performed using a portable ultrasonic spirometer in line with standard guidelines. Patients had to make a minimum of three attempts; the spirometer auto selects the best attempt and records of the forced expiratory volume in first second (FEV_1_), forced vital capacity (FVC), and FEV_1_/FVC. Impaired lung function was classified as obstructive (FEV_1_/FVC < 0.70), restrictive (FVC < LLN [lower limit of normal] with FEV_1_/FVC ≥ 0.70), PRISm (reduced FEV_1_ with normal FEV_1_/FVC and FVC), or combined (coexisting obstructive and restrictive defect). Any individual with either 6MWT < 400 m or impaired lung function was considered to have pulmonary disability.^30,31^
- Multimorbidity: Individuals with at least one comorbidity, risk determinant, or pulmonary disability were considered to have suffered from multimorbidity during the TB treatment.
- Referrals and linkage to care: Research staff facilitated onward referrals in accordance with pre-defined criteria (https://figshare.com/articles/presentation/Annexure_-1_Referral_and_Linkare_criteria_for_patients_completing_anti-TB_treatment/31890322?file=63286012). Linkage to care and start of care were assessed 1 month after treatment completion via telephone interviews with the patient.

### Data management and statistical analysis

Data were analysed using Stata v14 (StataCorp LP, College Station, TX, USA). Categorical variables were summarised as frequencies and percentages, while mean (standard deviation [SD]) or median (interquartile range [IQR]) was used to summarise continuous variables. Independent associations between demographic characteristics, risk determinants, comorbidities, and pulmonary disability were assessed using modified Poisson regression, and adjusted prevalence ratios (aPRs) with 95% confidence intervals (CI) were calculated.

### Ethical statement

This study was approved by the JIPMER Institutional Ethics Committee (JIP/IEC-OS/415/2023) and institutional ethics committees of the respective study sites. Permission was obtained from the district TB offices of all the study sites. Written informed consent was obtained from all participants.

## RESULTS

In total, 780 (67%) of 1,169 eligible patients were assessed for TB-associated disability. Of the 780 assessed, mean (SD) age was 42 years (16) and 58% were male. About 79% had pulmonary TB, 73% were bacteriologically confirmed, 92% were newly diagnosed, and 99% had drug-susceptible TB. The median (IQR) time taken to complete assessments was 29 min (20–37) (Table 1).

**TABLE 1.** Demographic and clinical characteristics of TB patients aged ≥ 18 years who successfully completed TB treatment and were assessed for comorbidities, risk determinants, and disability in selected primary health centres of India, 2024–2025.

Characteristics	n (%)
Total	780 (100)
District	
Puducherry	246 (31)
Chandigarh	52 (7)
Nagpur	138 (18)
Yadadri Bhongir	200 (26)
Deoghar	144 (18)
Age (in years)	
18–29	212 (27)
30–44	226 (29)
45–59	185 (24)
≥60	157 (20)
Sex	
Male	449 (58)
Female	331 (42)
Living area	
Urban	542 (70)
Rural	238 (30)
Site of TB	
Pulmonary	617 (79)
Extra-pulmonary	160 (21)
Both	3 (<1)
Type of TB	
Bacteriologically confirmed	572 (73)
Clinically diagnosed	208 (27)
Category of TB	
New	719 (92)
Previously treated	61 (8)
Drug-susceptibility	
Sensitive	776 (99)
RR/MDR-TB	4 (1)
Duration for assessment (in minutes)^A^	
<15	42 (5)
15–29	324 (42)
30–44	257 (33)
≥45	157 (20)

RR = rifampicin resistant; MDR = multidrug resistant.

A Time taken to perform assessments of comorbidities, risk determinants, and disability.

### Symptoms, comorbidities, risk factors, and disability

Of the 780 assessed, 180 (23%) had residual symptoms, with cough (n = 86; 11%) and shortness of breath (n = 84; 11%) being the most common. For comorbidities, 184 (24%) had DM/hyperglycaemia with newly detected hyperglycaemia in 23 (13%); 152 (19%) had high blood pressure with newly detected high blood pressure in 84 (55%); and 49 (6%) had a mental health disorder with newly detected ‘probable depression’ in 40 (82%). For risk determinants of TB, 281 (36%) had undernutrition, 16 (2%) had probable alcohol dependence, and 28 (4%) were using smoked tobacco (Table 2).

**TABLE 2.** On-going symptoms, comorbidities, risk determinants, and disability of TB patients aged ≥ 18 years who successfully completed TB treatment and were assessed for comorbidities, risk determinants, and disability in selected primary health centres of India, 2024–2025.

Variable	Categories	n (%)
Total		780 (100)
On-going symptoms		
Symptoms suggestive of TB	None	600 (77)
	Any symptom^A^	180 (23)
	Cough	86 (11)
	Shortness of breath	84 (11)
	Tiredness/fatigue	48 (6)
	Chest pain	30 (4)
	Other^B^	37 (5)
Comorbidities		
HIV status	Positive	06 (1)
	Negative	774 (99)
Diabetes mellitus (DM)/hyperglycaemia	Already known	161 (21)
	Tested for DM with RBG	584 (94)^C^
	Newly detected hyperglycaemia	23 (4)^D^
	Prevalent DM/hyperglycaemia (already known and new)	184 (24)
High blood pressure	Already known	68 (9)
	Newly detected	84 (12)^D^
	Prevalent high blood pressure (already known and new)	152 (19)
Mental health disorder	Already known	09 (1)
	Newly detected with probable depression	40 (5)^D^
	Prevalent mental health disorder (already known and new probable depression)	49 (6)
Risk determinants		
Probable alcohol dependence	CAGE score ≥ 2	16 (2)
Malnutrition	BMI < 18.5 kg/m^2^	281 (36)
Silica dust	Occupational exposure	12 (2)
Smoked tobacco	Anytime in last 1 month	28 (4)
Recreational drug use	Current use^E^	6 (1)
Pulmonary disability		
6-minute walk test (6MWT)	Done	667^F^ (86)
	6MWT < 400 m	165 (21)
Spirometry	Done	612^F^ (78)
	Impaired FEV_1_/FVC	367 (60)
Multimorbidity		
Comorbidity and/or risk determinant and/or disability	None	118 (15)
	One	237 (30)
	Two	219 (28)
	Three and above	206 (26)

RBG = random blood glucose; CAGE = Cut, Annoyed, Guilty, Eye questionnaire; BMI = body mass index; FEV_1_ = forced expiratory volume in first second; FVC = forced vital capacity.

A Some patients had multiple symptoms.

B Other symptoms such as fever, weight loss, night sweats, myalgia, joint pain, abdomen discomfort, and numbness of hands/feet.

C Percentages calculated with those not already known to have diabetes as denominator (N = 619).

D Percentages calculated with those who were not already known to have the condition and assessed for the condition as denominator.

E Recreational drugs included marijuana, trihexyphenidyl, and kuber (chewable nicotine).

F Only 667 and 612 patients underwent 6MWT and spirometry among 780 patients recruited, respectively.

Of the 780 patients, 6MWT was performed in 667 (85%), of which 165 (21%) walked < 400 m. 6MWT could not be conducted in those with weakness, lower limb ailments, or old age. Only 612 (78%) patients could perform spirometry, of which 367 (60%) had impaired FEV_1_/FVC with 268 (73%) having restrictive, 88 (24%) having obstructive, 4 (1%) having mixed, and 7 (2%) having PRISm pattern. To get successful reading on spirometry, it took median (IQR) attempts of 4 (3–6) and required multiple visits in 24 patients. Of the 753 patients who underwent either 6MWT or spirometry, 451 (60%, 95% CI: 56%–64%) had pulmonary disability. Overall, 667 (85%) patients had at least one comorbidity, risk factor, and/or disability (Table 2).

### Characteristics associated with pulmonary disability

On adjusted analysis, being aged ≥ 60 years (aPR-1.2, 95% CI: 1.0–1.4), having pulmonary TB (aPR: 1.3, 95% CI: 1.1–1.6), mental health disorder (aPR: 1.3, 95% CI: 1.1–1.5), undernutrition (aPR: 1.2, 95% CI: 1.1–1.4), smoked tobacco use (aPR: 1.4, 95% CI: 1.1-1.7), and occupational silica exposure (aPR: 1.4, 95% CI: 1.1–1.7) were independently associated with pulmonary disability (Table 3).

**TABLE 3.** Characteristics associated with pulmonary disability (6MWT < 400 m/deranged PFT) in TB patients aged ≥ 18 years who successfully completed TB treatment in selected health facilities in selected primary health centres of India, 2024–2025.

Variable	Total	Pulmonary disability	Crude	Adjusted
		n (%)	PR (95% CI)	PR (95% CI)
Total	753	451 (60)		
Age in years				
18–29	211	115 (55)	1	1
30–44	223	130 (59)	1.1 (0.9–1.3)	1.1 (0.9–1.3)
45–59	173	107 (62)	1.1 (0.9–1.3)	1.1 (0.9–1.3)
≥60	146	99 (68)	1.2 (1.1–1.5)	**1.2** (**1.0–1.4)**
Sex				
Male	431	262 (61)	1.0 (0.9–1.2)	0.9 (0.8–1.1)
Female	322	189 (59)	1	1
Site of TB				
Pulmonary	591	376 (64)	1.3 (1.1–1.6)	**1.3** (**1.1–1.6)**
Extra-pulmonary	159	75 (47)	1	1
Both	3	0 (0)	–	
Type of TB				
Bacteriologically confirmed	550	338 (61)	1.1 (1.0–1.3)	0.9 (0.8–1.0)
Clinically diagnosed	203	113 (56)	1	1
Category of TB				
New	694	411 (59)	1.1 (0.9–1.4)	1.1 (0.9–1.3)
Previously treated	59	40 (68)	1	1
Drug-susceptibility				
Sensitive	749	448 (60)	1	1
Resistant	4	3 (75)	1.3 (0.7–2.2)	1.3 (0.7–2.4)
HIV status				
Positive	6	5 (83)	1.4 (1.0–2.0)	1.4 (0.9–2.2)
Negative	747	446 (60)	1	1
Diabetes mellitus/hyperglycaemia^A^				
Yes	176	106 (60)	1.0 (0.9–1.1)	1.0 (0.8–1.2)
No	577	345 (60)	1	1
High blood pressure^B^				
Yes	142	86 (61)	1.0 (0.9–1.1)	1.0 (0.8–1.1)
No	611	365 (60)	1	1
Mental health disorder^C^				
Yes	46	36 (78)	1.3 (1.1–1.5)	**1.3** (**1.1–1.5)**
No	707	415 (59)	1	1
Probable alcohol dependence^D^				
Yes	14	6 (43)	0.7 (0.4–1.3)	0.6 (0.3–1.1)
No	739	445 (60)	1	1
Undernutrition^E^				
Yes	269	176 (65)	1.2 (1.0–1.2)	**1.2** (**1.1–1.4)**
No	484	275 (57)	1	1
Occupational exposure to silica				
Yes	12	11 (92)	1.5 (1.3–1.8)	**1.4** (**1.1–1.7)**
No	741	440 (59)	1	1
Smoked tobacco in the past month				
Yes	27	20 (74)	1.2 (1.0–1.5)	**1.4** (**1.1–1.7)**
No	726	431 (59)	1	1
Recreational drug use				
Yes	6	5 (83)	1.4 (1.0–2.0)	1.3 (0.8–2.4)
No	747	446 (60)	1	1
Multimorbidity (excluding 6MWT)				
None	254	136 (53)	1	
One	275	174 (63)	1.2 (1.0–1.3)	
Two	120	75 (62)	1.2 (1.0–1.4)	
Three and above	104	66 (63)	1.2 (1.0–1.4)	

The bold value signifies a significant association of pulmonary disability with respective variable with a *P* value of ≤0.05.

PR = prevalence ratio; 6MWT = six minute walk test; PFT = pulmonary function test; CI = confidence interval.

A Diabetes mellitus – Random blood glucose equal or greater than 200mg/dl.

B Hypertension – Systolic blood pressure ≥ 140mmHg or diastolic blood pressure ≥ 90mmHg.

C Mental health disorder – Patient Health Questionnaire-2 score of 3 or higher.

D Probable alcohol dependence – Cut, Annoyed, Guilty, Eye questionnaire score 2 or higher.

E Undernutrition – Body mass index < 18.5 kg/m^2^.

### Referral for care

For most conditions, 60%–100% were referred within the same facility, with the majority initiating care within a short duration (median 0–2 days). Notable exceptions included: 1) mental health disorders (12% referred) and 2) recreational drug use (33% referred, with no initiation of care) – see Table 4.

**TABLE 4.** Status of referral for further care in those identified with comorbidities, risk determinants, and disability among TB patients aged ≥ 18 years who successfully completed TB treatment in selected primary health centres of India, 2024–2025.

Conditions	Eligible for referral^A^	Referred to care	Referred within the same facility	Arrived at the referred facility	Initiated on care	Duration in days from referral to treatment initiation
	N	n (%)	n (%)^B^	n (%)^B^	n (%)^C^	Median (IQR)^D^
Comorbidities						
HIV	1	1 (100)	0 (0)	0 (0)	0 (0)	0 (0)
Diabetes mellitus/hyperglycaemia	37	23 (62.2)	18 (78.3)	11 (91.7)	8 (72.7)	2 (1–6)
Hypertension	122	83 (68.0)	67 (80.7)	51 (75.3)	33 (64.7)	1 (0–18)
Mental health disorder	43	5 (11.6)	3 (60.0)	3 (60.0)	3 (100.0)	7 (0–20)
Risk determinants						
Probable alcohol dependence	16	9 (56.2)	8 (88.9)	6 (66.7)	4 (66.7)	2 (0–20)
Undernutrition^E^	109	57 (52.3)	34 (64.9)	37 (76.0)	36 (97.3)	1 (0–6)
Occupational exposure to silica	12	5 (41.7)	5 (100.0)	2 (60.0)	1 (50.0)	5 (5–5)
Smoking	28	21 (75.0)	17 (80.9)	14 (73.7)	8 (57.1)	0 (0–3)
Recreational drug use	6	2 (33.3)	2 (100.0)	0 (0.0)	0 (0.0)	0 (0)
Disability						
Pulmonary disability^F^	59	51 (86.4)	30 (58.8)	29 (90.6)	22 (75.9)	2 (0–15)

IQR = interquartile range; BMI = body mass index; 6MWT = six minute walk test; FEV_1_ = forced expiratory volume in first second; FVC = forced vital capacity.

A For diabetes mellitus, hypertension, and mental health disorder, patients who were newly diagnosed and patients who were known to have the condition but not on care or had uncontrolled disease were considered eligible for referral.

B Percentages calculated with total referred to care which includes within the same facility or outside the same facility for the condition as denominator.

C Percentages calculated with total arriving at the referral facility as the denominator.

D Zero refers to initiation of treatment on the same day of the assessment.

E Only those with BMI < 16 kg/m^2^ were considered eligible for referral.

F Only those with 6MWT < 400 or impaired FEV_1_/FVC in spirometry and having Modified Medical Research Council Dyspnoea Score (mMRC) > 2 were eligible for referral.

### Work absenteeism

Among those employed or in school, 66% were absent for at least 1 day after TB diagnosis, due to symptoms severity (88%) and the fear of spreading infection (35%). The median (IQR) duration of absenteeism was 90 days (60–180) and wage loss was 495 USD (288–792). About 67% who were absent from work were family breadwinners (Table 5).

**TABLE 5.** Work absenteeism, reasons, duration of absenteeism, and wage loss during TB treatment among patients aged ≥ 18 years who successfully completed TB treatment and were assessed for comorbidities, risk factors, and disability in selected primary health centres of India, 2024–2025.

Characteristics	n (%)
Total^A^	508 (100)
Absent from work/school	
Yes	336 (66)
No	172 (34)
Reason for being absent (N = 328)^B^	
Severity of symptoms	296 (88)
To visit health facility	5 (2)
Advised to take sick leave by doctor	37 (11)
Patient’s fear of spreading infection	117 (35)
Compulsory sick leave from workplace	35 (10)
Others	11 (3)
Median (IQR) days of absenteeism after TB diagnosis	90 (60–180)
Median (IQR) wage loss (in USD) due to absenteeism among those who were absent^C^ (N = 291)	495 (288–792)
Whether the patient who was absent from work was the primary bread winner of the family (N = 291)	
Yes	194 (67)
No	97 (33)

IQR = interquartile range.

A Includes those who were employed (163), self-employed (127), a daily wage labourer (133), and a student (86).

B Several patients had multiple reasons for being absent and numbers do not add up to 508. Multiple reasons are possible and the total percentage does not add up to 100%.

C Includes those who are employed (163), self-employed (127), and a daily wage labourer (133).

## DISCUSSION

This study from five Indian districts highlights the burden of TB-associated disability among those completing TB treatment. The study has five key findings. First, four out of five patients had multimorbidity. Second, three out of five patients had pulmonary disability. Third, the prevalence of undernutrition, DM, and hypertension was high, with majority of those having hypertension and mental health disorders being newly diagnosed during assessment, highlighting that this screening is worthwhile. Fourth, referral and linkage to care were suboptimal. Finally, work absenteeism was reported by two thirds of patients with an average wage loss of about 500 USD during TB treatment.

The high prevalence of post-TB disability underscores the fact that after completing treatment and achieving a bacteriological cure, the majority of people have lung impairment. Previous spirometry studies from single tertiary care facilities in India also reported a high burden (38%–63%) of pulmonary disability.^4,18–20^ In China (21%) and Africa (20%), where only 6MWT was used, the prevalence was relatively low and comparable to that found using 6MWT in the current cohort (21%).^14–17^ With regard to spirometry, pulmonary disability is classified as individuals having structural damage in the lungs with or without functional disability, whereas the 6MWT is focused exclusively based on functional disability.^32,33^ A patient with a 30% reduction in lung volume is considered to have lung impairment in spirometry, but may walk at a normal pace for 6 min without hitting their physiological limit, and appear ‘normal’ on the 6MWT.^32^ Because spirometry is difficult to perform and requires multiple attempts, the 6MWT may be a better option for assessing functional pulmonary disability in the routine programme setting.^32^ However, future studies are needed to generate evidence on the most appropriate tools and algorithms for assessing pulmonary disability.

A higher multimorbidity (85%) was seen in the current study compared to the Africa cohort (78%).^16,17^ Though the prevalence of risk determinants (probable alcohol dependence, smoked tobacco use, and silica exposure) was lower, the high prevalence of comorbidities (pulmonary disability, DM, and hypertension) led to a higher prevalence of multimorbidity. Although the prevalence of DM was high, most patients already knew their DM status and were on treatment, as NTEP has successfully integrated DM screening at the start of TB treatment.^13^ In contrast, the majority of hypertension and mental health disorder were newly detected at the end of TB treatment, indicating lost opportunities to detect and manage it during TB treatment. Unidentified comorbidities may delay recovery and negate long-term survival in TB patients.^14–17^ Similar to findings from Africa, those who were biologically vulnerable (old, undernourished, and having mental health disorder) and exposed to pulmonary irritants (smoked tobacco and silica exposure) had higher risk of pulmonary disability.^16,17^ Old age, undernutrition, and mental health disorder could reduce strength and performance of respiratory muscles, whereas pulmonary irritants can cause inflammation and fibrosis leading to structural lung damage.^9,14,17,20^ Thus, interventions targeting associated risk factors early during the treatment would reduce pulmonary disability. This is substantiated by a recent study from Africa, which showed a significant reduction in TB-associated disability including pulmonary disability when patient-centred assessment and management of risk determinants, comorbidities, and pulmonary disability are integrated at the start of TB treatment.^34^

Though the research staff attempted to refer individuals with TB-associated disability for further treatment, referral and linkage remained suboptimal, in contrast to high rates in Africa where health staff conducted the assessments in the health facilities.^16,17^ As research staff might be perceived as outsiders of the health system, patients might have declined referrals and may not have adhered to their advice on future care. Thus, it would be logical to embed these assessments within routine TB care services and strengthen the management of TB-associated disability. As the assessments could be completed within 30 min in the majority of patients, integration with routine care services may not substantially increase the workload of health staff.^15^

One third of the patients with TB reported work absenteeism with loss of income and reduced productivity.^16,17,35^ This indicates that TB extends beyond a health issue and has significant social and economic implications due to wage loss and associated stigma. As 67% of patients were primary breadwinners, financial instability could prevail within the family leading to increased catastrophic expenditure and delayed health seeking, thereby worsening the disease outcomes.^35^ This highlights the need to strengthen social protection during TB treatment.

The study strengths included a multi-centric approach lending generalisability, large sample size, use of spirometry in addition to 6MWT, and adoption of standardised protocol enabling cross-country comparison. Not embedding the project within the routine programmatic setting was a major limitation as it impacted the coverage of assessment and referral mechanisms and may not represent feasibility in the routine care setting.

## CONCLUSION

The prevalence of multimorbidity, including pulmonary disability, is high among those completing TB treatment, highlighting that bacteriological cure does not guarantee a productive life with full recovery. To enhance health-related quality of life in TB patients, assessment and management of TB-associated disability at both the start and end of TB treatment should be instituted within routine TB services.
